# The AMPAR antagonist perampanel protects the neurovascular unit against traumatic injury via regulating Sirt3

**DOI:** 10.1111/cns.13580

**Published:** 2021-01-09

**Authors:** Tao Chen, Wen‐Bo Liu, Xiao Qian, Ke‐Liang Xie, Yu‐Hai Wang

**Affiliations:** ^1^ Department of Neurosurgery The 904th Hospital of PLA Medical School of Anhui Medical University Wuxi China; ^2^ Translational Research Institute of Intensive Care Medicine College of Anesthesiology Weifang Medical University Weifang China; ^3^ Department of Anesthesiology Tianjin Research Institute of Anesthesiology Tianjin Medical University General Hospital Tianjin China; ^4^ Department of Critical Care Medicine Tianjin Research Institute of Anesthesiology Tianjin Medical University General Hospital Tianjin China

**Keywords:** AMPA receptor, neurovascular unit, perampanel, Sirt3, traumatic brain injury

## Abstract

**Introduction:**

Perampanel is a highly selective and noncompetitive α‐amino‐3 ‐hydroxy‐5‐methyl‐4‐isoxazole propionate receptor (AMPAR) antagonist, which has been used as an orally administered antiepileptic drug in more than 55 countries. Recently, perampanel was shown to exert neuroprotective effects in hemorrhagic and ischemic stroke models via regulating blood–brain barrier (BBB) function.

**Aim:**

Here, the protective effects of perampanel were investigated in an in vitro neurovascular unit (NVU) system established using a triple cell co‐culture model (neurons, astrocytes, and brain microvascular endothelial cells) and in an in vivo traumatic brain injury (TBI) model.

**Results:**

Neurons in the NVU system exhibit a more mature morphological phenotype compared with neurons cultured alone, and the co‐culture system mimicked an impermeable barrier in vitro. Perampanel protects the NVU system against traumatic and excitotoxic injury, as evidenced by reduced lactate dehydrogenase (LDH) release and apoptotic rate. Treatment with perampanel attenuated lipid peroxidation and expression of inflammatory cytokines. In addition, perampanel increased Sirt3 protein expression, enhanced the activities of mitochondrial enzyme IDH2 and SOD2, and preserved BBB function in vitro. Knockdown of Sirt3 using specific siRNA (Si‐Sirt3) partially reserved the effects of perampanel on neuronal injury and BBB function. Treatment with perampanel in vivo attenuated brain edema, preserved neurological function, inhibited apoptosis and microglia activation after TBI. Furthermore, perampanel increased the expression of Sirt3 and preserved BBB function after TBI. The effect of perampanel on BBB function and brain edema was abolished by knockdown of Sirt3 in vivo.

**Conclusion:**

Our results indicate that the noncompetitive AMPAR antagonist perampanel protects the NVU system and reduces brain damage after TBI via activating the Sirt3 cascades.

## 1 INTRODUCTION

Traumatic brain injury (TBI) refers to a disruption in the normal function of the brain caused by external forces, and it affects more than 50 million people each year in the whole world.^1^ However, the molecular mechanisms of neuronal damage and neurological deficits following TBI are not fully determined. It is currently accepted that TBI should be considered as not a single pathophysiological event, but a cascade that involves complex signaling pathways.^2^, ^3^, ^4^, ^5^ In the process of TBI, it has been shown that brain microvascular endothelial cells (BMEC) and astrocytes support neurons to form the neurovascular unit (NVU), which is a complex structural and functional unit with protective effects on the blood–brain barrier (BBB).^6^, ^7^, ^8^, ^9^, ^10^ The NVU becomes a new target for TBI research in recent years.^11^, ^12^, ^13^, ^14^, ^15^

Glutamate is one of the key excitatory neurotransmitters in the brain and the over‐release of glutamate and over‐activation of its receptors are considered to be involved in TBI‐induced neuronal injury.^16^ Perampanel is a novel noncompetitive antagonist of the α‐amino‐3‐hydroxy‐5‐methyl‐4‐isoxazole propionate (AMPA) receptor (AMPAR), a subtype of the ionotropic glutamate receptors (iGluRs). Many in vivo and in vitro experimental studies and clinical trials have demonstrated that perampanel exerted antiepileptic properties by modulating glutamatergic synaptic excitation, and it has been proved to be used for the treatment of partial‐onset seizures.^17^ Recently, some experimental data indicated that perampanel could exert neuroprotective effects in various neurological disorders, including intraventricular hemorrhage and ischemic stroke.^18^, ^19^, ^20^ Our previous study showed that perampanel protects against brain damage following TBI in rats via both anti‐oxidative and anti‐inflammatory activity.^21^ In addition, Lv et al. showed that perampanel exerts neuroprotective effects via the claudin‐5 mediated modulation of BBB permeability in ischemic stroke models.^22^ However, the effects of perampanel on NVU system in vitro and BBB function after TBI, as well as the potential underlying molecular mechanisms have not been investigated. Here, we investigated the effect of perampanel on NVU system in vitro and BBB function after TBI in vivo, and we also determined the potential molecular mechanisms with focus on the Sirt3 signaling pathway.

## 2 MATERIALS AND METHODS

### 2.1 Animals

All experimental animals, including six pregnant female Sprague–Dawley rats (E16‐18), eight male Sprague–Dawley rats (2–3 day old), and eight male Sprague–Dawley rats (10 day old), were obtained from the Animal Experimental Center of Anhui Medical University.

### 2.2 Establishment of the NVU system

**FIGURE 1:**
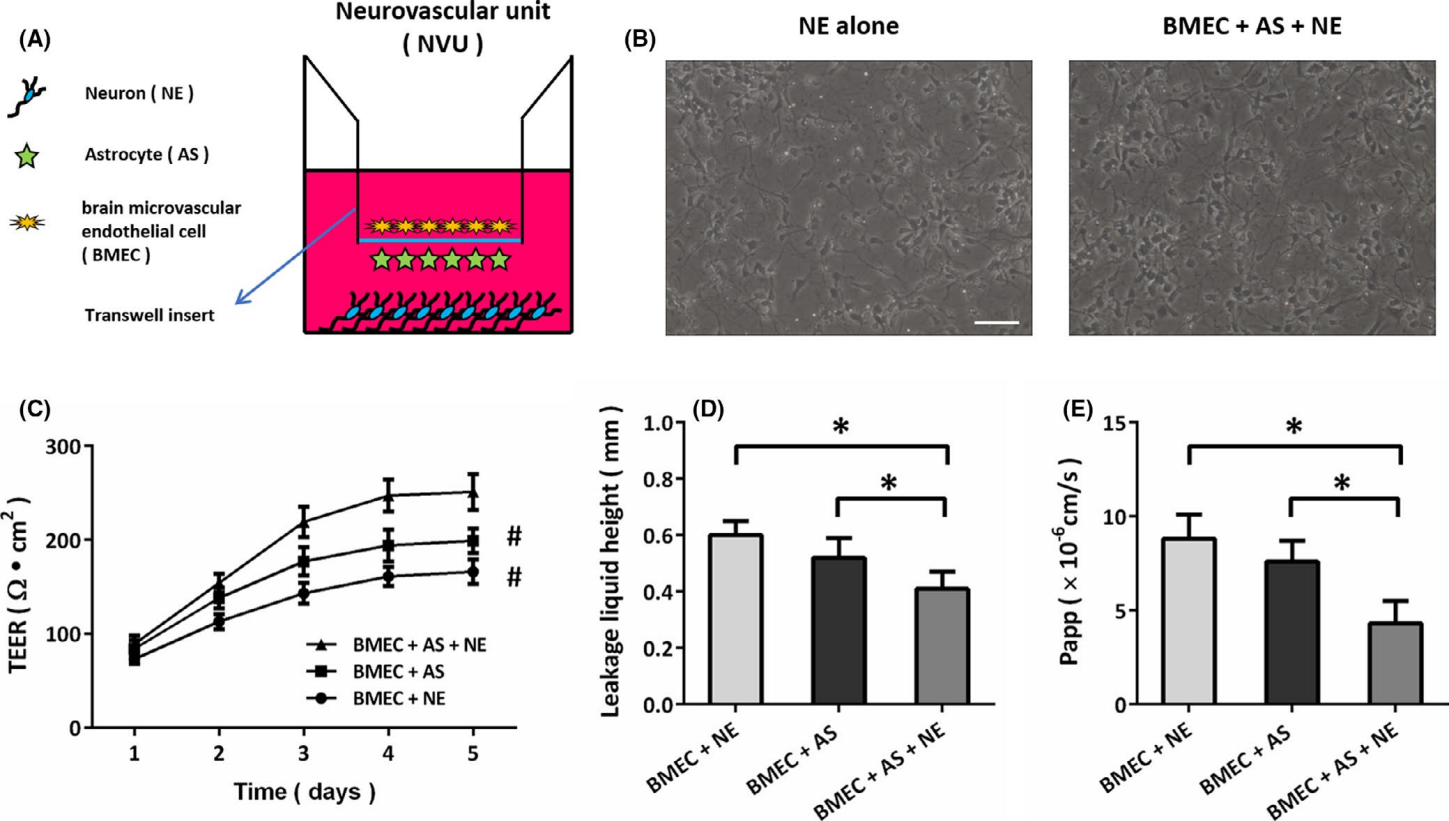
Morphological and functional features of the NVU model in vitro. (A) Diagrams of the in vitro NVU system. (B) Neurons (NE) co‐cultured with astrocytes (AS) and brain microvascular endothelial cells (BMEC) exhibit a more mature morphological phenotype, as evidenced by increased soma volume and axonal width. (C) NE co‐cultured with AS and BMEC show a higher TEER value. (D) NE co‐cultured with AS and BMEC show a lower leakage liquid height. (E) NE co‐cultured with AS and BMEC show a lower Papp value. Scale bar, 50 μm. Error bars indicate SEM (*n* = 6). ^#^ *p* < 0.05 vs. BMEC+AS+NE group. *p < 0.05

Cortical neurons were cultured from pregnant female Sprague–Dawley rats (E16‐18) using our previously described methods.^23^ Astrocytes were cultured from eight male Sprague–Dawley rats (2–3 day old), and BMEC were cultured from eight male Sprague–Dawley rats (10 day old) as previously described.^24^ As shown in Figure 1A, our in vitro NVU system was established using a triple cell co‐culture model (neurons, astrocytes, and BMEC) as previously described.^24^

### 2.3 In vitro models

To investigate the effect of perampanel in our NVU system in vitro, the triple cell co‐culture model was exposed to traumatic or excitotoxic insult. The traumatic neuronal injury (TNI) model, which was performed only on neurons, was established using a rotating scribe device as we previously reported.^25^ In addition, the NVU was exposed to 100 μM glutamate to induce excitotoxicity in vitro. Perampanel was purchased from Santa Cruz (sc‐477647, CA, USA), and the cells were treated with perampanel at the beginning of TNI or glutamate insult.

### 2.4 Transendothelial electrical resistance (TEER) measurement

TEER was measured using a resistance meter (ERS‐2, Millipore, Billerica, MA, USA). The overall resistance can characterize the formation of a tight endothelial cell monolayer. The co‐cultures were allowed to equilibrate at 25°C for 20 minutes before detection. The TEER of the inserts was calculated by subtracting the resistance of the control insert from that of the insert and multiplying the difference by the area of the insert.

### 2.5 Sodium fluorescein permeability measurement

We evaluated the apparent permeability (Papp) for the small molecule sodium fluorescein (SF; 376 Da) to detect the formation of tight junctions by BMEC. SF at the concentration of 100 μg/ml was added in the upper chamber. A 100 μl volume was collected from the lower chamber at 15, 30, 45, and 60 min, and then replenished with pre‐equilibrated culture media. The absorbances of the samples were assayed by a fluorescence spectrophotometer. Papp = (d*M*/d*t*)/(*A*·*C*), where d*M*/d*t* is the cumulative measured fluorescence intensity in the plate per unit time *A* refers to the bottom area of the insert (1.12 cm^2^), and *C* refers to the SF intensity in the upper insert.

### 2.6 Lactate dehydrogenase (LDH) release assay

To assay the cellular injury in vitro in the NVU system, LDH release into the culture medium was determined using a commercial kit according to the manufacture's protocol (Ji‐Di‐Ao, Shanghai, China). Briefly, 50 µl of supernatant from each well was collected, incubated with reduced form of nicotinamide‐adenine dinucleotid (NADH) and pyruvate for 15 min at 37°C and the reaction was stopped by adding 0.4 M NaOH. The activity of LDH was calculated from the absorbance at 440 nm and background absorbance from culture medium that was not used for any cell cultures was subtracted from all absorbance measurements.

### 2.7 Hoechst staining

After perampanel and various treatments as described earlier, neurons were stained with 10 µg/ml Hoechst 33342 to detect the condensed nucleus,^26^ a key marker for apoptotic cells.

### 2.8 Measurement of lipid peroxidation

Malonyl dialdehyde (MDA) and 4‐hydroxynonenal (4‐HNE), two index of lipid peroxidation, were detected by commercial assay kits according to the manufacturer's instruction (Jian‐Cheng Biotech, Nanjing, China).

### 2.9 Measurement of inflammatory cytokines

The concentrations of IL‐1β and IL‐6in different time points were measured using specific ELISA kits from Boster Biological Technology according to the manufacturers’ protocols.

### 2.10 Short interfering RNA (siRNA) and transfection

The specific siRNA targeted Sirt3 (Si‐Sirt3, sc‐61556) and control siRNA (Si‐control, sc‐37007) were purchased from Santa Cruz. For in vitro experiments, the siRNA molecules were transfected using Lipofectamine RNAiMax reagent (Invitrogen) in Opti‐MEM medium according to the manufacturer's instructions. For in vivo experiments, animals were anesthetized in the stereotaxic frame, and a midline scalp incision was made. Five microliters of the siRNA molecules were injected into the right lateral ventricle at a rate of 0.5 μl/min. The rats were subjected to TBI at 72 h after the injection.

### 2.11 TBI model

TBI was induced by using a controlled cortical impact (CCI) model in according with previously detailed methods.^21^ The rats were orally administered with saline water or perampanel (5 mg/kg) at 5 min after TBI.

### 2.12 Measurement of brain water content

Brain edema was determined with the wet‐dry method 24 h after TBI. Brain water content was then calculated using the following formula: % H_2_O = (1−dry weight/wet weight) × 100%.

### 2.13 Measurement of neurological function

Neurological function was evaluated using a 6‐point scoring system at 24 h after TBI. For appetite: 0, finished meal; 1, left meal unfinished; 2, scarcely ate. For activity: 0, walk and reach at least three corners of the cage; 1, walk with some stimulation; 2, almost always lying down. For deficits: 0, no deficits; 1, unstable walk; 2, impossible to walk.

### 2.14 TUNEL staining

Apoptosis in brain sections was determined by measuring DNA fragmentation using a standard TUNEL staining method according to the manufacturer's protocol (Roche, Penzberg, Germany). In brief, sections of 4 μm thick were cut, mounted on poly‐L‐lysine‐coated slides, and treated with proteinase K solution (20 μg/ml) for 10 min at room temperature to permeabilize tissues. TUNEL staining was performed by labeling with fluorescein TUNEL reagent mixture for 60 min at 37°C according to the manufacturer's suggested protocol and examined under a fluorescence microscopy. The number of TUNEL‐positive cells in each section in 10 microscopic fields (at ×600 magnification) was counted by an investigator blinded to the grouping.

### 2.15 Immunofluorescent staining

Brain sections with 4 μm thickness (fixed with 4% paraformaldehyde) were treated with 0.1% Triton X‐100 and then were blocked by 5% bovine serum albumin (BSA). The samples were incubated with the Iba‐1 or Sirt3 primary antibody at 4°C overnight. After being washed by phosphate‐buffered saline with Tween‐20 (PBST) for three times, the samples were incubated with the secondary antibodies at 37°C for 1 h. Then, 4,6‐diamidino‐2 ‐phenylindole (DAPI) was used to stain the nuclei, and the pictures were obtained using a Zeiss fluorescent imaging microscope (Carl Zeiss, Thornwood, NY, USA).

### 2.16 Western blot analysis

The cell homogenates were obtained from the mixed culture with 3 types of cells. A standard Western blot assay was performed using the following primary antibodies: Sirt3 (#2627, Cell Signaling, 1:1000) and β‐actin (ab8226, Abcam, 1:5000). After incubation with secondary antibodies for 1 h, the bands were visualized by using chemiluminescent detection system.

### 2.17 Statistical analysis

Statistical analysis was performed using the GraphPad Prism 6.0. Statistical evaluation of the data was analyzed using Student's t test or randomized one‐way ANOVA, followed by Tukey's post hoc test, to compare the differences between groups.

## 3 RESULTS

### 3.1 Morphological and functional features of the NVU model in vitro

The in vitro NVU model was established using the co‐culture system of neurons, astrocytes, and BMEC as shown in Figure 1A. Neurons in the NVU system exhibit a more mature morphological phenotype, as evidenced by increased soma volume and axonal width, as compared to neurons cultured alone (Figure 1B). The NVU model reached a maximum TEER at 5 days, which was significantly higher than the values in BMEC co‐cultured either with neurons or with astrocytes (Figure 1C). We measured the leakage liquid height to detect the permeability of the NVU system, and the results showed that BMEC co‐cultured with neurons and astrocytes exhibited the lowest permeability (Figure 1D). In addition, we measured the permeability coefficient of sodium fluorescein (SF), and the low paracellular Papp of SF in the NVU system suggested the formation of an impermeable barrier in vitro (Figure 1E).

### 3.2 Perampanel protects NVU against traumatic and excitotoxic injury

**FIGURE 2:**
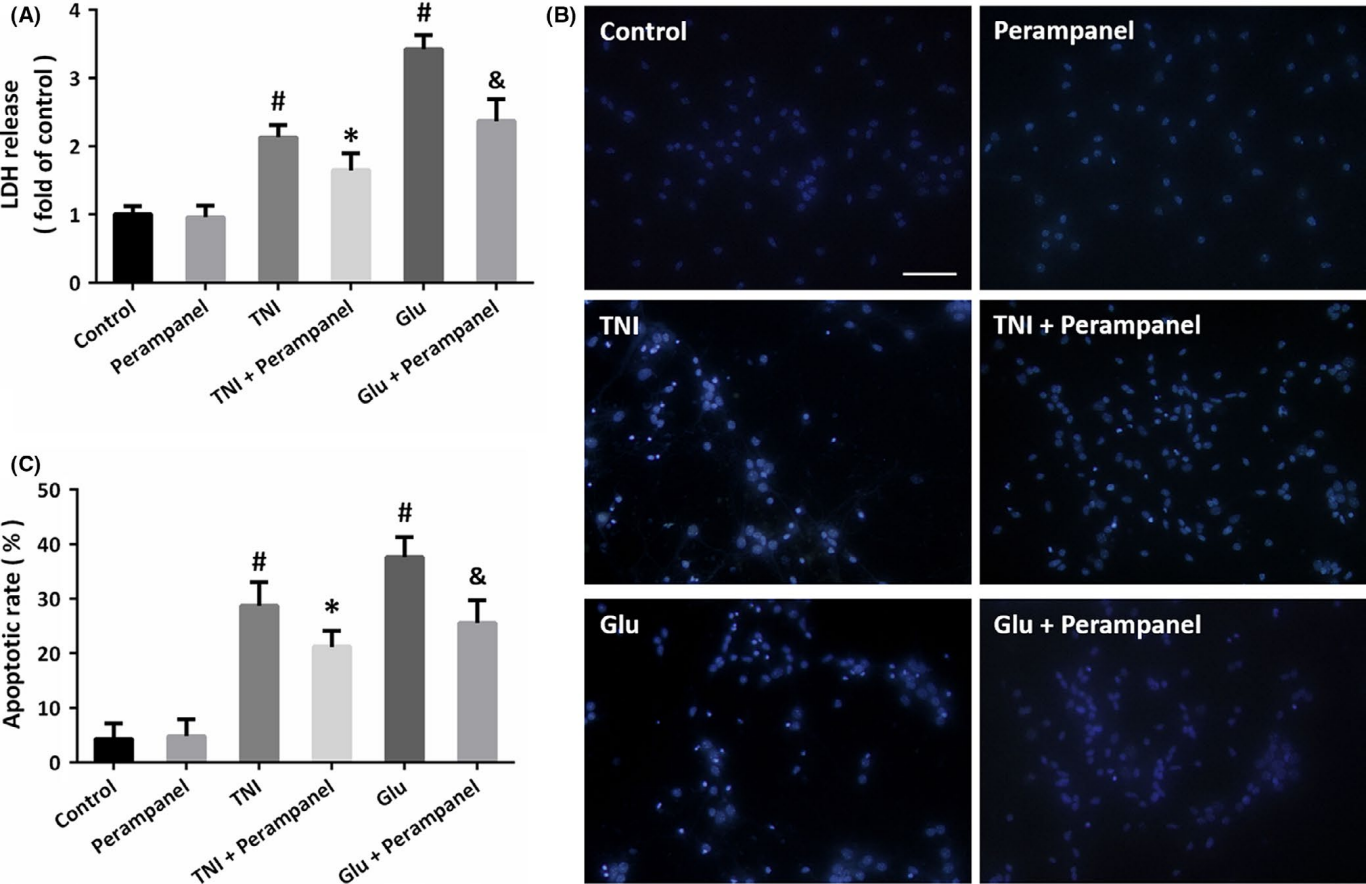
Perampanel protects NVU against traumatic and excitotoxic injury. (A) Perampanel attenuates the LDH release induced by traumatic neuronal injury (TNI) or glutamate (Glu) in cultured cortical neurons at 24 h. (B and C) Hoechst staining (B) and quantification (C) show that perampanel inhibits neuronal apoptosis after TNI and glutamate treatment at 24 h. Scale bar, 50 μm. Error bars indicate SEM (*n* = 6). ^#^ *p* < 0.05 vs. Control group. *p < 0.05 vs. TNI group. ^&^ *p* < 0.05 vs. Glu group

To mimic the neuronal injury induced by TBI in vitro, the NVU system was treated with TNI or glutamate. The results of LDH release assay showed that TNI and glutamate significantly increased the LDH release, which were both attenuated by perampanel at 24 h (Figure 2A). We performed the Hoechst staining to detect the apoptotic cell death in neurons at 24 h (Figure 2B), and the results showed that the increased apoptotic rate induced by TNI and glutamate were alleviated by perampanel treatment (Figure 2C).

### 3.3 Perampanel attenuates oxidative stress and neuroinflammation

**FIGURE 3:**
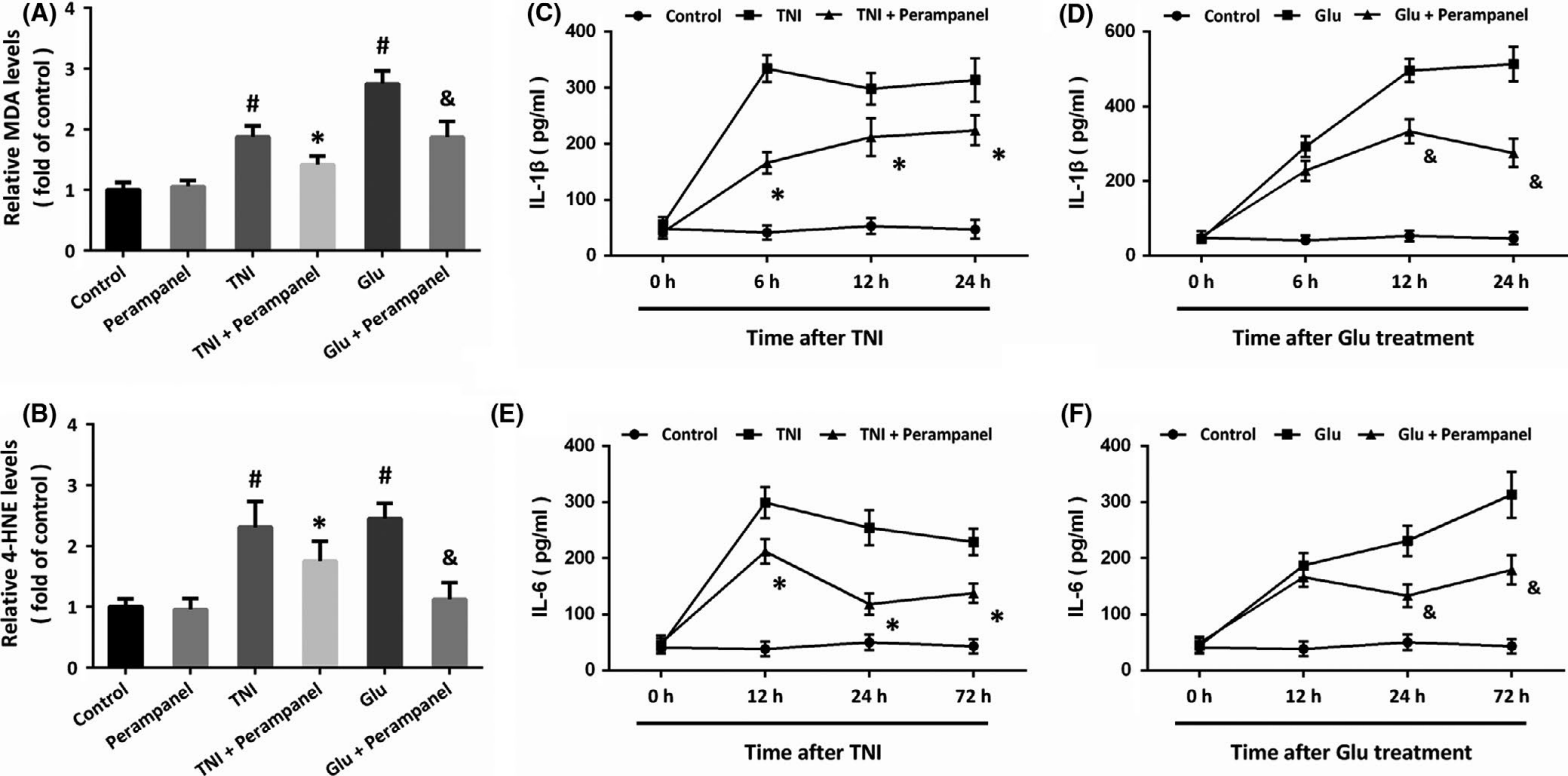
Perampanel attenuates oxidative stress and neuroinflammation. (A and B) Perampanel inhibits lipid peroxidation, as evidenced by reduced levels of MDA (A) and (4‐HNE). (C and D) Perampanel reduces IL‐1β level after TNI (C) and glutamate treatment (D). (E and F) Perampanel reduces IL‐6 level after TNI (E) and glutamate treatment (F). Error bars indicate SEM (*n* = 6). ^#^ *p* < 0.05 vs. Control group. *p < 0.05 vs. TNI group. ^&^ *p* < 0.05 vs. Glu group

**FIGURE 4:**
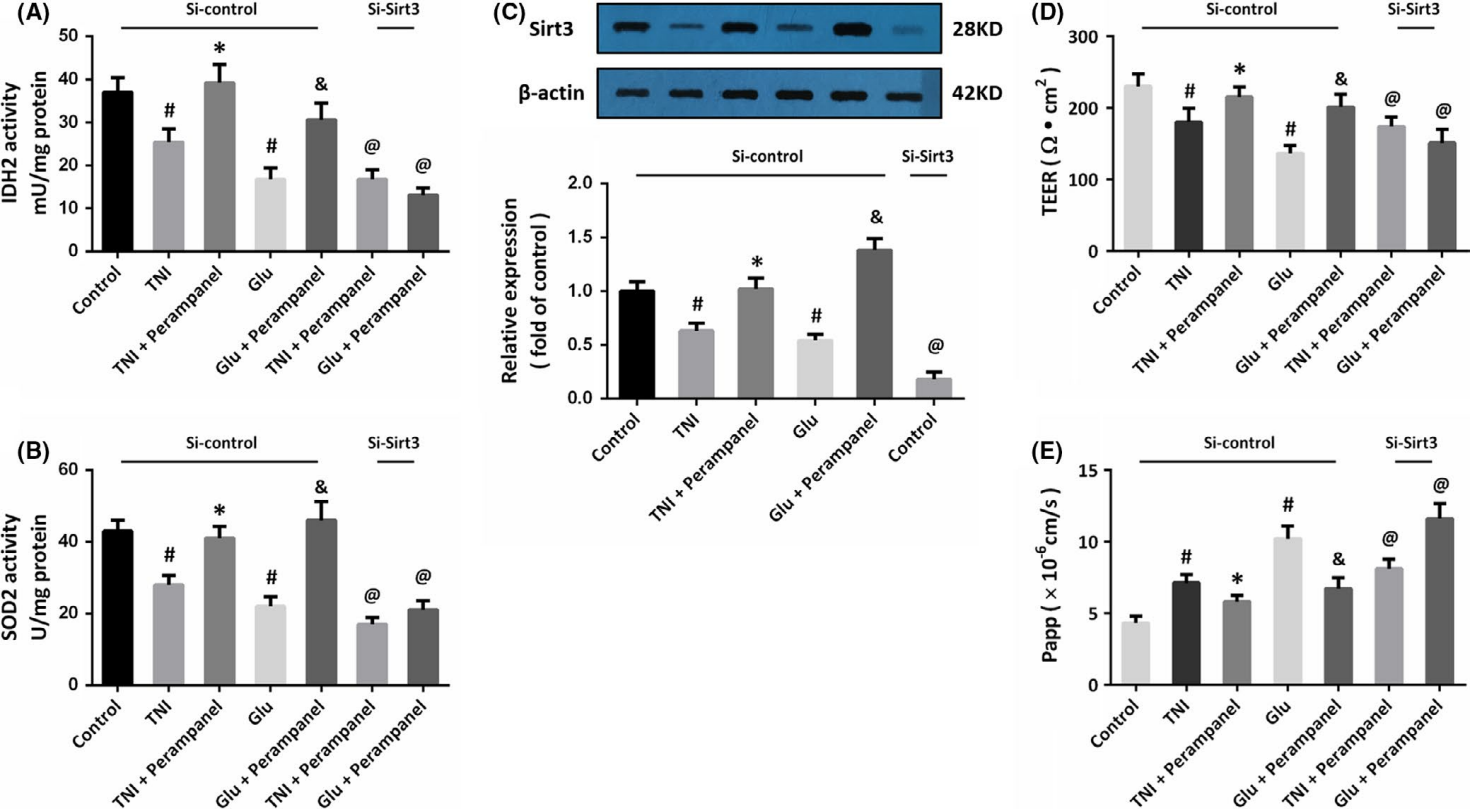
Perampanel preserves BBB function via Sirt3 in the NVU. (A and B) Perampanel preserves the enzymatic activities of IDH2 (A) and SOD2 (B) via activating Sirt3. (C) Perampanel increases Sirt3 expression after TNI or glutamate treatment. (D and E) Perampanel preserves BBB function, as evidenced by increased TEER value (D) and decreased Papp value (E), after TNI or glutamate treatment via activating Sirt3. Error bars indicate SEM (*n* = 6). ^#^ *p* < 0.05 vs. Control group. *p < 0.05 vs. TNI group. ^&^ *p* < 0.05 vs. Glu group. ^@^ *p* < 0.05 vs. Si‐control group

Next, we detected the levels of MDA (Figure 3A) and 4‐HNE (Figure 3B), two indicators of lipid peroxidation, to investigate the effect of perampanel on oxidative stress in the NVU system. The results showed that TNI and glutamate treatment increased the levels of MDA and 4‐HNE, which were both attenuated by perampanel. The increased level of IL‐1β induced by TNI at 6, 12, and 24 h were decreased by perampanel (Figure 3C), while perampanel reduced IL‐1β level at 12 and 24 h after glutamate treatment (Figure 3D). In addition, the increased level of IL‐6 induced by TNI at 6, 12, and 24 h were decreased by perampanel (Figure 3E), whereas perampanel reduced IL‐6 level at 12 and 24 h after glutamate treatment (Figure 3F).

### 3.4 Perampanel preserves BBB function via Sirt3 in the NVU

We measured the enzymatic activity of IDH2 (Figure 4A) and SOD2 (Figure 4B), two key anti‐oxidative mitochondrial enzymes. The results showed that the activities of these enzymes were inhibited by TNI and glutamate, but perampanel preserved the enzymatic activities of IDH2 and SOD2 in the NVU system. The results of Western blot showed that perampanel significantly increased the expression of Sirt3 after TNI or glutamate treatment (Figure 4C). Transfection with specific siRNA (Si‐Sirt3) was performed to downregulate the expression of Sirt3 in our in vitro model. Knockdown of Sirt3 abolished the effect of perampanel on IDH2 and SOD2 activities (Figure 4A,B). Furthermore, perampanel markedly preserved the BBB function of the NVU system after TNI or glutamate treatment, as evidenced by the preserved TEER (Figure 4D) and the reduced paracellular Papp of SF (Figure 4E). However, all the effects of perampanel on TEER and Papp after TNI or glutamate treatment were reversed by knockdown of Sirt3.

### 3.5 Involvement of Sirt3 in perampanel‐induced protection

**FIGURE 5:**
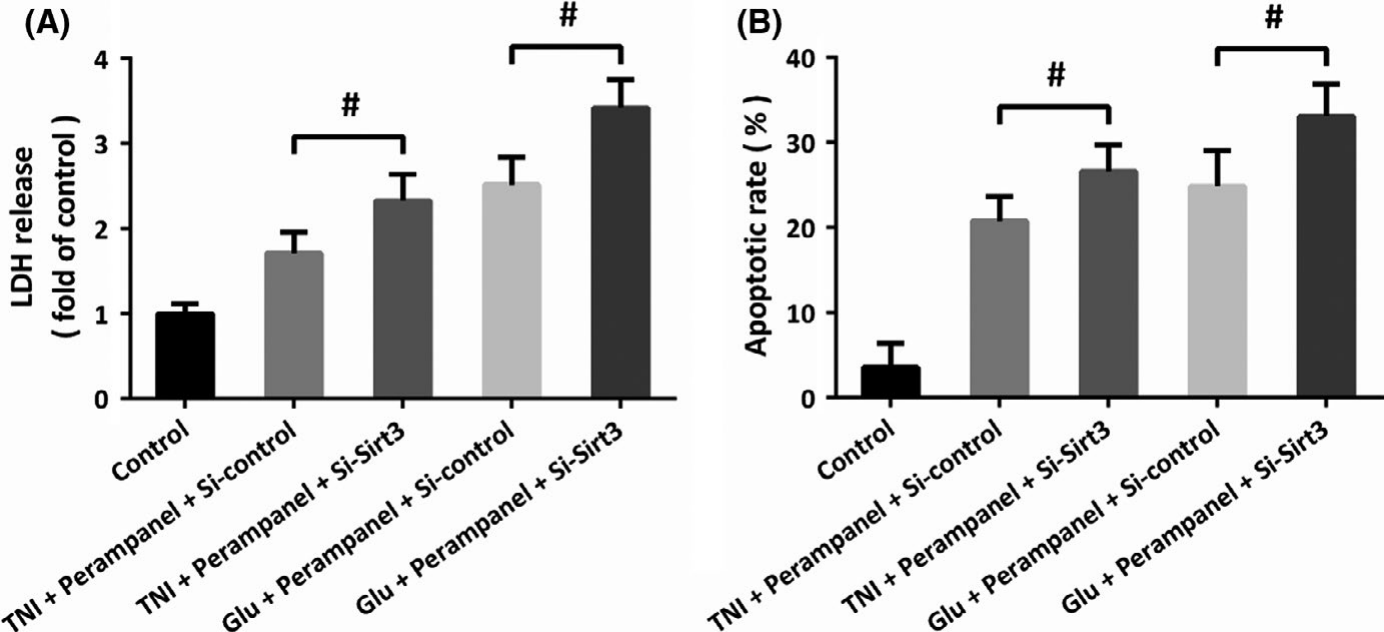
Involvement of Sirt3 in perampanel‐induced protection. (A) The perampanel‐induced decrease in LDH release after TNI or glutamate treatment was prevented by Si‐Sirt3 transfection. (B) The perampanel‐induced decrease in apoptotic rate after TNI or glutamate treatment was prevented by Si‐Sirt3 transfection. Error bars indicate SEM (*n* = 6). ^#^ *p* < 0.05

To further confirm the role of Sirt3 in perampanel‐induced protection in our in vitro model, we repeated the LDH release assay and TUNEL staining assay after Si‐Sirt3 transfection. The results of LDH release assay showed that the decreased LDH release induced by perampanel after TNI and glutamate treatment were both prevented by knockdown of Sirt3 (Figure 5A). As shown in Figure 5B, a similar result of apoptotic rate was also observed.

### 3.6 Perampanel protects against TBI‐induced brain damage and neuroinflammation

**FIGURE 6:**
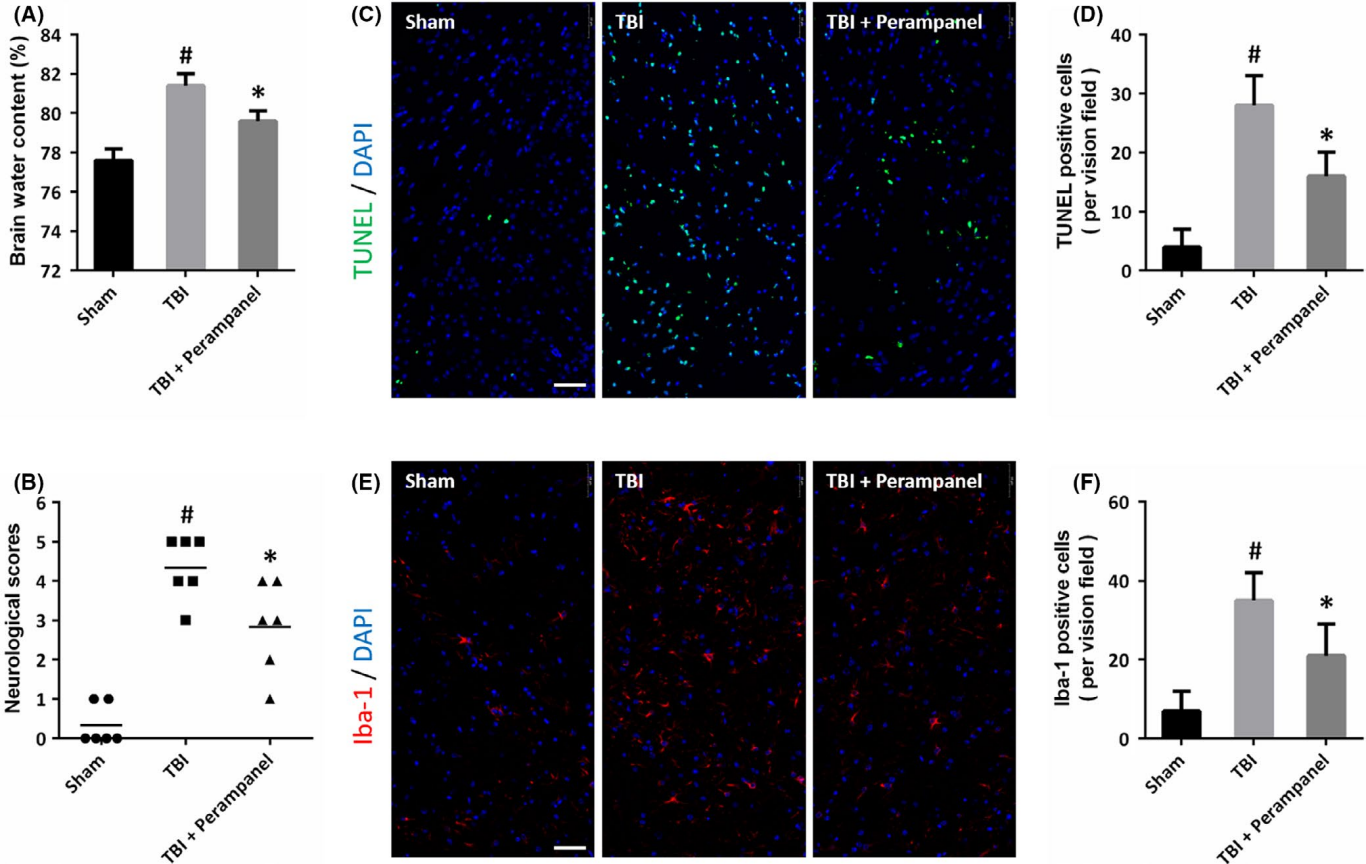
Perampanel protects against TBI‐induced brain damage and neuroinflammation. (A) Perampanel decreases brain water content at 24 h after TBI. (B) Perampanel preserves neurological function at 72 h after TBI. (C and D) TUNEL staining (C) and quantification (D) show that perampanel inhibits neuronal apoptosis at 24 h after TBI. (E and F) Immunostaining (E) and quantification (F) show that perampanel inhibits the activation of microglial cells at 24 h after TBI. Scale bar, 50 μm. Error bars indicate SEM (*n* = 6). ^#^ *p* < 0.05 vs. Sham group. *p < 0.05 vs. TBI group

To investigate the effect of perampanel in vivo, animals were injured by TBI and treated with perampanel. The results of brain water content showed that perampanel reduced brain edema after TBI at 24 h (Figure 6A). Perampanel also preserved the neurological function after TBI, as evidenced by reduced neurological scores at 72 h (Figure 6B). We also performed TUNEL staining in brain sections (Figure 6C), and the results showed that perampanel inhibited apoptosis at 24 h after TBI (Figure 6D). In addition, the activation of microglial cells was detected by immunostaining using Iba‐1 antibody (Figure 6E), and the results showed that perampanel attenuated the TBI‐induced activation of microglial cells at 24 h (Figure 6F).

### 3.7 Perampanel regulates BBB function via Sirt3 after TBI

**FIGURE 7:**
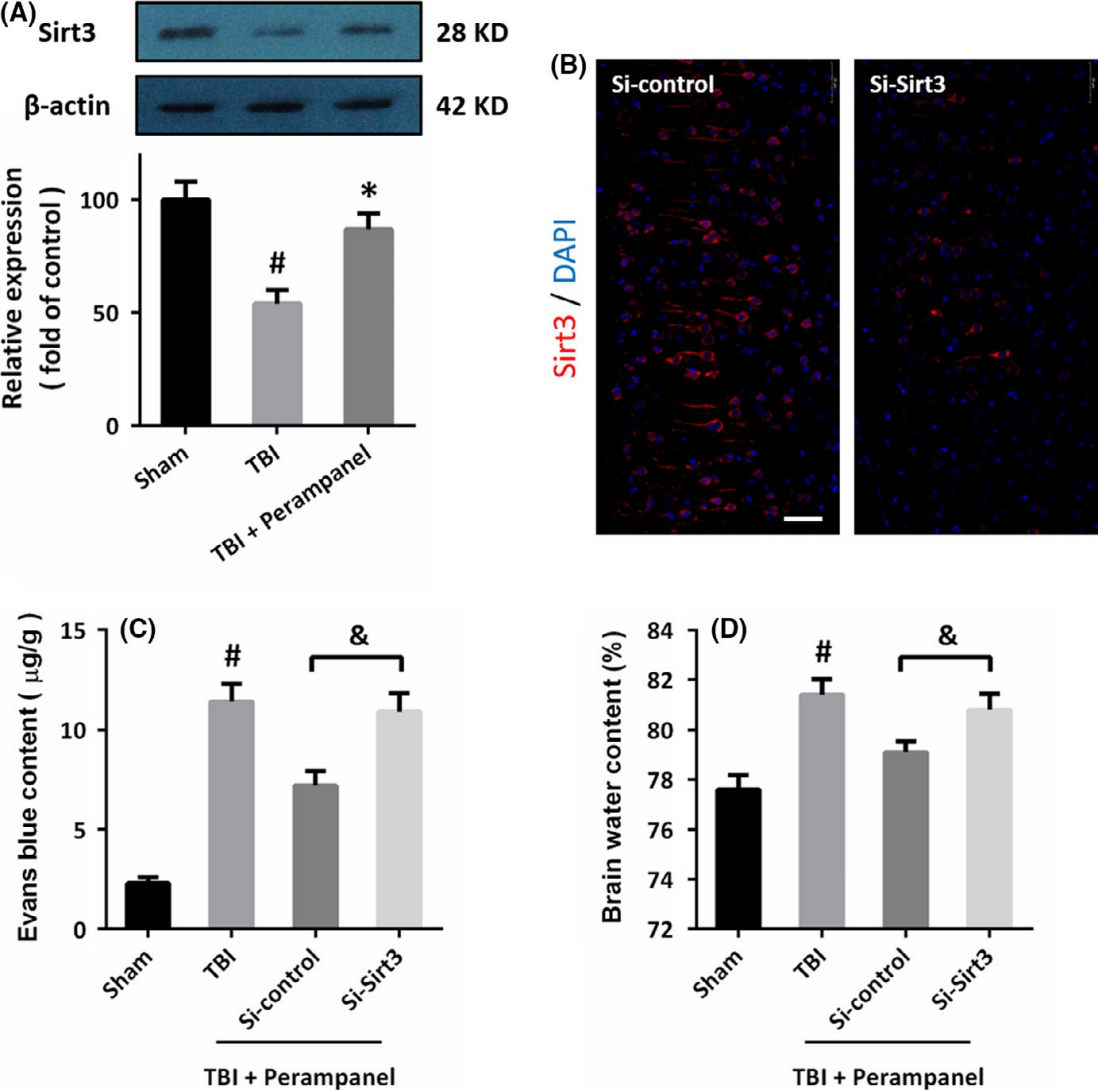
Perampanel regulates BBB function via Sirt3 after TBI. (A) Perampanel preserves Sirt3 expression after TBI. (B) Transfection with Si‐Sirt3 decreases the expression of Sirt3 in vivo. (C) Perampanel decreases Evans blue content via Sirt3 after TBI. (D) The perampanel‐induced decrease in brain water content after TBI was prevented by Sirt3 knockdown. Scale bar, 50 μm. Error bars indicate SEM (*n* = 6). ^#^ *p* < 0.05 vs. Sham group. *p < 0.05 vs. TBI group. ^&^ *p* < 0.05

The results of Western blot showed that TBI decreased the expression of Sirt3 in the brain, but perampanel increased Sirt3 expression after TBI (Figure 7A). To further investigate the role of Sirt3 in our in vivo model, we infused Si‐Sirt3 into the right cortex of rats to downregulate the expression of Sirt3, and the results showed that Si‐Sirt3 significantly decreased Sirt3 expression in brain sections (Figure 7B). The BBB function in vivo was assayed by measuring Evans blue content, and the results showed that perampanel decreased Evans blue content after TBI, which was partially prevented by Sirt3 knockdown (Figure 7C). Also, the perampanel‐induced protection on brain edema after TBI was alleviated by Sirt3 knockdown (Figure 7D).

## 4 DISCUSSION

The noncompetitive AMPAR antagonist perampanel has been demonstrated to exert neuroprotective effects against intraventricular hemorrhage, ischemic stroke, and TBI by many previous studies. The present study identified perampanel as a neuroprotective agent that attenuates brain damage and neuronal injury following TBI via Sirt3‐mediated preservation of NVU function. We found that (a) perampanel protects NVU against traumatic and excitotoxic injury in vitro; (b) perampanel attenuates oxidative stress and neuroinflammation; (c) perampanel preserves BBB function via Sirt3 in the NVU; (d) perampanel protects against TBI‐induced brain damage and neuroinflammation; and (e) perampanel regulates BBB function via Sirt3 after TBI in vivo.

Perampanel is an allosteric inhibitor of AMPAR with high selectivity for receptor binding to AMPAR versus N‐methyl‐d‐aspartate (NMDA) receptors (NMDAR), another subtype of the iGluRs. A previous study has shown that the IC_50_ of perampanel for inhibiting AMPAR‐induced calcium currents was 93 nM in primary cultured neurons.^27^ In congruent, perampanel could reduce AMPAR‐evoked excitatory postsynaptic field potentials with an IC_50_ of 0.23 μM and a full blockage at 3 μM.^28^ Thus, 3 μM perampanel was used in our in vitro model, and significant protective effects were observed by morphological and cell toxicity assays. In a recent in vitro study using primary brain endothelial cell cultures, perampanel was found to preserve the BBB function after ischemia at the concentration of 3 μM.^22^ Perampanel can be rapidly absorbed from the gastrointestinal tract with a bioavailability of 100%, and data from various animal species demonstrate that it is widely distributed to tissues, including the brain.^29^ For patients, perampanel is available as tablets in 2–12 mg doses,^29^ and the concentration of perampanel used in our experiments can be easily obtained after oral administration. To date, perampanel has been licensed as monotherapy or adjunctive therapy for some types of epilepsy in patients over 12 years in more than 55 countries, including the USA and China,^30^ making it an ideal candidate for the drug research of other neurological disorders.

The [^3^H]‐AMPA binding activity cannot be observed when perampanel was used at the dose of 1.25 μM, whereas the [^3^H]‐perampanel binding activity to neuronal membrane in rat brain cannot be disturbed even treated with high concentrations of AMPA, indicating the noncompetitive feature of perampanel to AMPAR.^27^ As neuroprotective agents under excitotoxic conditions, the noncompetitive antagonists were thought to have more advantages over the competitive antagonists, partially due to the effectiveness of noncompetitive antagonists at high agonist concentrations.^31^ A previous study demonstrated that perampanel could prevent the increased brain endothelial cell permeability after ischemia in vitro,^22^ which was consistent with previous study showing the effective BBB penetration of perampanel.^32^ In the present study, we successfully established an in vitro NVU system using the triple cell co‐culture model (neurons, astrocytes, and BMEC). The structure, function, and neuronal morphology of the NVU system were measured and compared with two other different double cell co‐culture models (BMEC and astrocytes, or BMEC and neurons). The higher TEER, lower leakage liquid height, and paracellular Papp of SF, as well as a more mature morphological phenotype of neurons, indicated the formation of an impermeable barrier in vitro. In this system, our data showed that the TNI‐ or glutamate‐induced decrease in TEER and increase in Papp were all prevented by perampanel, which were accompanied by reduced oxidative stress and expression of inflammatory cytokines. In addition, the protective effects of perampanel on BBB function after TBI were also observed in the in vivo model. All these results strongly indicate that preservation of the NVU structure and function contribute to perampanel‐induced protection against TBI.

The sirtuins, a conserved class of histone deacetylases (HDACs) that named after the founding member Sir2‐ins, have seven mammalian family members known as Sirtuin 1 to 7 (Sirt1 to Sirt7).^33^ Sirt3 is a mitochondrial deacetylase with high expression levels in highly metabolic tissues, including the brain, and has been shown to exert protective effects under stress conditions.^34^, ^35^, ^36^ The hyperacetylation‐related dysfunction of oxidative phosphorylation and fatty acid β‐oxidation, as well as insulin resistance and obesity, was found in Sirt3 knockout mice.^37^ Sirt3 has been demonstrated to limit reactive oxygen species (ROS) generation via deacetylate SOD2 (also known as MnSOD) and IDH2 in various neuronal cells.^38^, ^39^ Under stress conditions, Sirt3 could attenuate apoptosis by inhibiting ROS production and blocking the opening of the mitochondrial permeability transition pore (mPTP).^34^, ^40^ We have previously demonstrated that Sirt3 protects HT22 cells against the hydrogen peroxide‐induced oxidative stress via inhibition of mitochondrial dysfunction.^41^ By using the lentivirus transfection technology, we overexpressed Sirt3 protein in primary cultured cortical neurons and found that the overexpressed neurons are less vulnerable to oxidative stress with higher mitochondrial calcium regulating capacity and mitochondrial biogenesis.^42^ In line with our results, Sirt3 was recently shown to directly deacetylase and stabilize 8‐oxoguanine‐DNA glycosylase 1 (OGG1) to promote mitochondrial DAN (mtDNA) repair.^43^ More importantly, using an in vitro ischemia model, we demonstrated that downregulation of Sirt3 could aggravate the oxygen‐glucose deprivation‐induced BBB disruption and apoptosis.^44^ In this study, we found that the perampanel‐induced protection in BBB function, as evidenced by TEER and Papp in vitro, as well as Evans blue content in vivo, were all partially reversed by Sirt3 knockdown. Furthermore, these effects were accompanied by the changes in oxidative stress and neuroinflammation, the downstream cascades of BBB breakdown. In congruent, Sirt3 was demonstrated to be beneficial to neurovascular and functional recovery following chronic ischemic stroke.^45^ Thus, activation of Sirt3 might be one of the molecular mechanisms that mediate perampanel‐induced protection against TBI.

There are some limitations to this study. First, the NVU is a complex structural and functional unit. It consists of BMEC, neurons, astrocytes, microglial cells, and the extracellular matrix.^46^ In this study, the NVU system was established using a triple cell co‐culture model (neurons, astrocytes, and BMEC), and the role of microglial cells and extracellular matrix has not been investigated. In addition, it is well‐known that TBI is one of the leading causes of morbidity and mortality in adult people. In this study, embryonic cortical neurons were used to investigate the effect of perampanel in vitro. Some more experiments using more mature neurons are needed in future.

## 5 CONCLUSION

In summary, our present data demonstrated that the noncompetitive AMPAR antagonist perampanel protects the NVU system against traumatic and excitotoxic injury in vitro. Perampanel attenuated brain damage inhibited neuroinflammation and preserved BBB function in an in vivo TBI model. All these protective effects were found to be mediated by the activation of Sirt3. Some more experiments are needed to determine the role of perampanel in microglial cells and extracellular matrix following TBI.

## CONFLICT OF INTEREST

The authors declare no conflicts of interest.

## Supporting information

Figure S1

## Data Availability

The data used to support the findings of this study are available from the corresponding author upon request.
