# Supplementary figures and images for: The AMPAR antagonist perampanel protects the neurovascular unit against traumatic injury via regulating Sirt3
Source: CNS Neurosci Ther. 2021 Jan 9;27(1):134–44. doi: 10.1111/cns.13580 (PMC7804923; doi:10.1111/cns.13580)

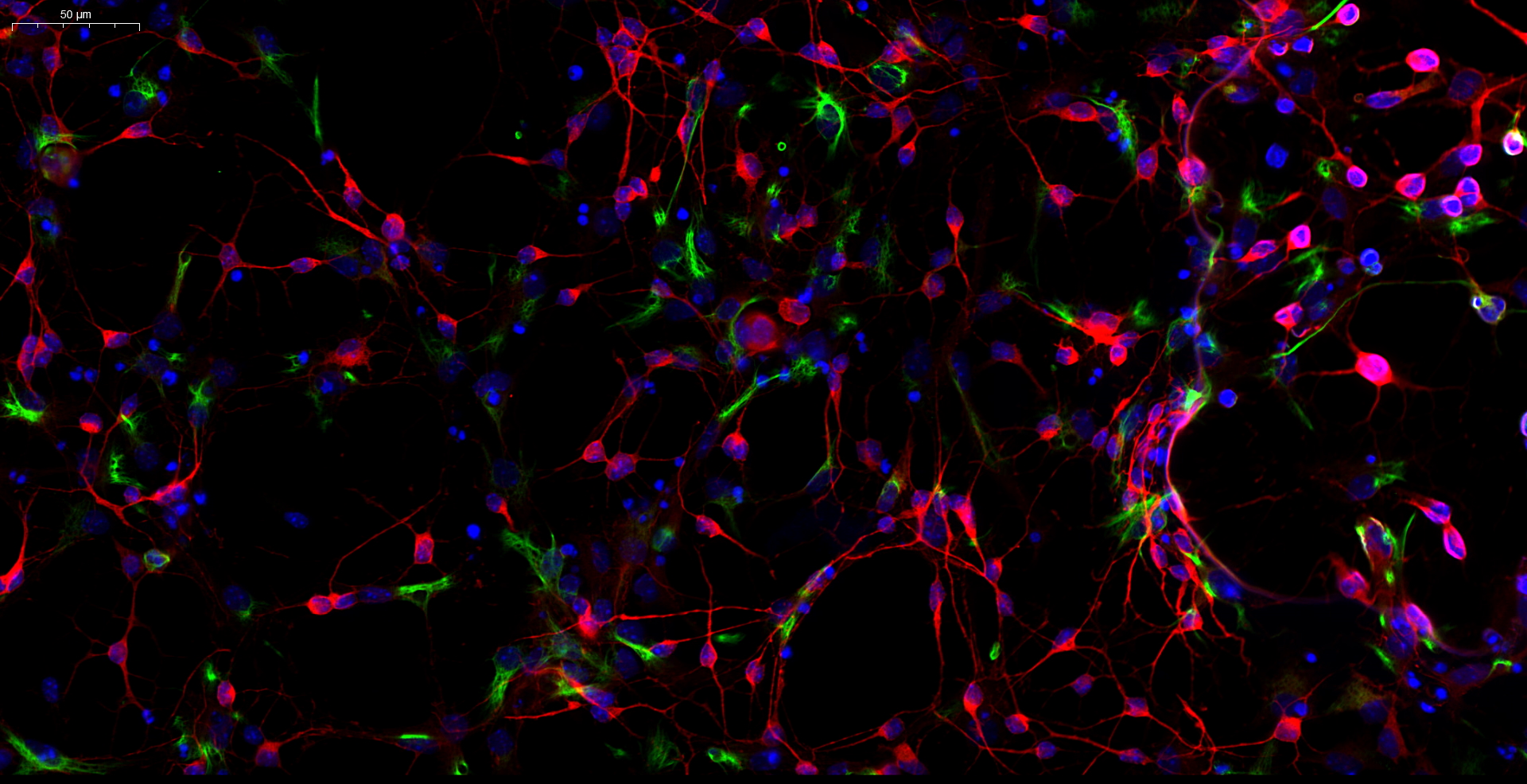

Supplement: Supplementary file 1 — Figure S1 [file CNS-27-134-s001.jpg]
